# *CRISP2*, *CATSPER1* and *PATE1* Expression in Human Asthenozoospermic Semen

**DOI:** 10.3390/cells10081956

**Published:** 2021-07-31

**Authors:** Francesco Manfrevola, Bruno Ferraro, Carolina Sellitto, Domenico Rocco, Silvia Fasano, Riccardo Pierantoni, Rosanna Chianese

**Affiliations:** 1 Dipartimento di Medicina Sperimentale, Sez. Bottazzi, Università degli Studi della Campania “L. Vanvitelli”, Via Costantinopoli 16, 80138 Napoli, Italy; francesco.manfrevola@unicampania.it (F.M.); roccodomenico01@gmail.com (D.R.); silvia.fasano@unicampania.it (S.F.); riccardo.pierantoni@unicampania.it (R.P.); 2 UOSD di Fisiopatologia Della Riproduzione, Presidio Ospedaliero di Marcianise, 81025 Caserta, Italy; brunoferraro1@virgilio.it (B.F.); carolinasellitto@hotmail.com (C.S.)

**Keywords:** circRNAs, mRNAs, sperm motility, asthenozoospermia, sperm quality

## Abstract

The etiology of human asthenozoospermia is multifactorial. The need to unveil molecular mechanisms underlying this state of infertility is, thus, impelling. Circular RNAs (circRNAs) are involved in microRNA (miRNA) inhibition by a sponge activity to protect mRNA targets. All together they form the competitive endogenous RNA network (ceRNET). Recently, we have identified differentially expressed circRNAs (DE-circRNAs) in normozoospermic and asthenozoospermic patients, associated with high-quality (A-spermatozoa) and low-quality (B-spermatozoa) sperm. Here, we carried out a differential analysis of *CRISP2*, *CATSPER1* and *PATE1* mRNA expression in good quality (A-spermatozoa) and low quality (B-spermatozoa) sperm fractions collected from both normozoospermic volunteers and asthenozoospermic patients. These sperm fractions are usually separated on the basis of morphology and motility parameters by a density gradient centrifugation. B-spermatozoa showed low levels of mRNAs. Thus, we identified the possible ceRNET responsible for regulating their expression by focusing on circTRIM2, circEPS15 and circRERE. With the idea that motility perturbations could be rooted in quantitative changes of transcripts in sperm, we evaluated circRNA and mRNA modulation in A-spermatozoa and B-spermatozoa after an oral amino acid supplementation known to improve sperm motility. The profiles of CRISP2, CATSPER1 and PATE1 proteins in the same fractions of sperm well matched with the transcript levels. Our data may strengthen the role of circRNAs in asthenozoospermia and shed light on the molecular pathways linked to sperm motility regulation.

## 1. Introduction

Human ejaculate consists of heterogeneous pools of spermatozoa varying in characteristics such as shape, size and motility that are conveyed along the epididymis through the seminal plasma, which is the non-cellular liquid component of semen that is a great source of biomarkers related to sperm quality [1,2,3]. High-quality spermatozoa (arbitrarily called A-spermatozoa) show convenient abilities related to fertilization, such as (i) normal morphology, (ii) good motility and (iii) the absence of DNA fragmentation [4,5]. On the other hand, the key features of low-quality spermatozoa (arbitrarily called B-spermatozoa) are high DNA fragmentation, morphological defects and low motility. In the case of male infertility, A-spermatozoa are properly selected for Assisted Reproduction Techniques (ART) procedures [6], with methods dictated by the nature of the semen sample and developed to mimic some of the natural selection processes that exist in the female reproductive tract [2,3,7]. In detail, sperm separation can be carried out by using the swim up technique or the density gradient centrifugation. The first one allows a lower recovery of motile spermatozoa (<20%) with a greater level of non-sperm components [8] than the density-gradient centrifugation (>20%), even if it possesses higher DNA integrity [2,6,9]. Conversely, in the cases of severe oligozoospermia, teratozoospermia or asthenozoospermia, the density gradient centrifugation is considered the preferred technique to select the major number of motile spermatozoa with a low risk of dead sperms or bacterial contaminations [2,8,10,11,12].

Asthenozoospermia is a condition of male infertility that is characterized by absent or reduced sperm motility [13]. This pathology is defined as a decrease in total motility (<40%) and progressive motility (<32%) occurring in the semen samples [2]. Sperm motility strongly depends on the functionality of mitochondria, which are the organelles rearranged at the level of the sperm flagellum midpiece, are able to convert chemical energy into mechanical energy and critical are for sperm quality [14,15,16]. However, alternative metabolic pathways have been hypothesized to contribute to the total ATP production and, therefore, to sperm motility [16,17].

Pathogenic factors for asthenozoospermia are complex and multiple and, thus, the molecular mechanisms underlying this state of infertility have not yet been fully elucidated despite the progress made in related research.

In this scenario, transcriptomic analysis has revealed a differential expression of thousands of genes in spermatozoa collected from fertile and infertile individuals [18], suggesting that sperm RNA profiling may be indicative of sperm quality and fertility status. In the context of differentially expressed (DE)-genes related to asthenozoospermia, special attention has recently been focused on Cysteine-Rich Secretory Protein 2 (*CRISP2*), Cation Channel Sperm Associated 1 (*CATSPER1*) and Prostate and Testis Expressed 1 (*PATE1*) [19,20,21,22,23]. Their preferential localization in sperm flagellum and their significant reduction in asthenozoospermic patients indicate that they are important targets to be validated [20,21,24,25,26]. In detail, the analysis of two *CRISP2*-deficient mouse lines defines a role for CRISP2 in sperm motility since its loss causes a shift toward slower motility and the stiff midpiece syndrome [27]. This depends on the CRISP2 ability to regulate calcium flow through ryanodine receptors [28] and its ability to be a CATSPER1 binding protein [27]. CATSPER1, as a voltage-gated calcium permeable channel specifically expressed on the plasma membrane of a sperm tail, is essential for sperm motility and hyperactivation through the regulation of calcium concentration [26,29]. The antibody blocking strategy points to PATE1 as another modulator of sperm motility [20]. The analysis of polymorphisms in *PATE1* gene suggested that its variant is a high risk genetic factor for human idiopathic asthenozoospermia [23]. Accordingly, knockout animal models strongly confirm a functional link between these modulators and sperm motility. *CRISP2* knockout mice showed fertility disorders, especially due to lower levels of sperm hyperactivation, the vigorous motility required for penetration of the egg coats [30]. Similarly, sperm motility and fertilization ability were markedly decreased in *CATSPER* knockout mice [29,31].

Circular RNAs (circRNAs)—covalently closed RNAs produced by back-splicing reactions—are especially involved in microRNAs (miRNAs) inhibition by a sponge activity [32,33,34,35]. This molecular action protects the mRNA targets of miRNAs from degradation and, thus, forms a complex network of endogenous RNAs, also known as the competitive endogenous RNA (ceRNA) network (ceRNET). CircRNAs have been identified in human testis and in mouse spermatogenic cells [35,36,37]. In rats, they exhibit higher tissue specificity than cognate mRNAs with a dynamic pattern in the testis, which is characterized by a dramatic increase with advancing stages of sexual maturity and a decrease with aging [38]. In human testis, an altered profile of circRNAs has been reported in the case of non-obstructive azoospermia [39].

Until today, few suggestions about circRNA role in sperm quality have been proposed. Only recently and by using a microarray strategy, a specific pattern of DE-circRNAs has been identified in both normozoospermic and asthenozoospermic patients that is associated with good and low quality sperm [40,41]. The circRNAome in ejaculated porcine sperm has also been characterized, finding significant correlations between the abundance of 148 exonic circRNAs and sperm motility parameters [42]. Accordingly, in humans, the motility improvement after the pharmacological treatment of asthenozoospermic patients completely reverted sperm derived circRNA cargo [41].

Based on this background, in the current study, we carried out a differential analysis of *CRISP2*, *CATSPER1* and *PATE1* mRNA expression separately in A-spermatozoa and B-spermatozoa of both normozoospermic volunteers and asthenozoospermic patients in order to evaluate their specific profile correlated with the motility parameter.

In order to assess a possible regulatory network upstream of *CRISP2*, *CATSPER1* and *PATE1* mRNAs, we pointed to circRNAs as integral parts of ceRNET by using a bioinformatic approach and by taking advantage of our previous microarray data.

With the idea that motility perturbations could be rooted in quantitative changes of transcripts in sperm, we evaluated circRNA and mRNA modulation in A-spermatozoa and B-spermatozoa derived from asthenozoospermic patients after the pharmacological treatment known to improve sperm motility.

Similarly, the profile of CATSPER1, PATE1 and CRISP2 proteins was also assessed in order to provide insights in the molecular mechanism connecting the different amounts of mRNAs and sperm motility control.

Our data may enrich the new findings concerning the role of circRNAs in asthenozoospermia and shed light on the molecular pathways linked to sperm motility regulation.

## 2. Materials and Methods

### 2.1. Human Semen Samples

The Marcianise Hospital Unit-UOSD of Physiopathology of Reproduction provided semen samples of normozoospermic and asthenozoospermic (*n* = 20) patients. Semen samples were produced by masturbation after 5–7 days of sexual abstinence and collected in sterile sample containers. After liquefaction for 30 min at 37°C, sperm samples were analyzed to evaluate semen parameters, such as concentration, total motility, progressive motility and morphology by using computer-assisted sperm analysis (CASA) technology associated with the Sperm Class Analyzer (SCA) system (SCA version 6.1; Microptic, S.L. Viladomat, Barcelona, Spain) and implemented with several software modules (SCA^®^ Motility and Concentration; SCA^®^ Morphology; SCA^®^ Vitality), in accordance to the WHO reference criteria. The microscope used with SCA was a Nikon Eclipse E200 with a X10 phase objective; samples were analyzed under negative phase contrast. The analysis was carried out by using a Makler^®^ counting chamber and the images were captured by using a digital camera Basler (75 fps) with a capture time of 1 s/field.

Sperm morphology parameters were analyzed by the SCA^®^ Morphology software module, which reported the percentage of head defects, midpiece defects, tail defects and cytoplasmatic droplets.

Sperm motility parameters were analyzed by SCA^®^ Motility and Concentration software module that, by calculating several kinematic parameters, provided the percentage of total and progressive motility of sperm sample.

In addition, an aliquot of all sperm samples was used to assess sperm vitality by Trypan blue staining (Trypan Blue, 0.4% Solution, 17-942E Lonza). Microscope analysis confirmed that only dead cells were positive for Trypan blue staining, whereas motile spermatozoa were not marked (data not shown).

### 2.2. Ethical Approval

In accordance with the Declaration of Helsinki, sperm samples were obtained from normozoospermic volunteers and asthenozoospermic patients after obtaining written informed consent. This study involving human participants was reviewed and approved by the ethics committee of Azienda Sanitaria Locale (ASL) Caserta, Regione Campania (n. 1353 del 27 October 2017). All patients were interviewed in order to better understand their area of origin, their eating habits, as well as their lifestyles.

### 2.3. Spermatozoa Isolation by Density Gradient Centrifugation

Human spermatozoa were purified by using a 40/80% discontinuous PureCeption (Cooper Surgical, Trumbull, CT, United States) centrifugation gradient. Each gradient was prepared in a conical plastic tube 30 mm in diameter by placing 1 mL of 80% PureCeption solution at the base and 1 mL of 40% PureCeption solution at the top of the tube. Subsequently, 1 mL of human semen sample was loaded for each PureCeption gradient, at the top of gradient and centrifuged at 300× *g* for 20 min. Following centrifugation, seminal plasma was removed. From 40% PureCeption abnormal spermatozoa (B-spermatozoa; B-SPZ) fraction was purified, while, from 80% PureCeption, a good motile spermatozoa (A-spermatozoa; A-SPZ) fraction was purified. The two sperm fractions were then washed once with 10 mL of sperm washing medium (HTF-IrvineScientific^®^) to remove the PureCeption and then centrifuged at 500× *g* for 15 min. Following centrifugation, an aliquot of the sample was used to evaluate the number of live and motile spermatozoa and to exclude dead cell contamination. The analysis of live spermatozoa was performed under a light microscope by using the viable dye Trypan-blue and counting the percentage of live/total spermatozoa, while the analysis of motile spermatozoa was performed by counting the percentage of motile/live spermatozoa (data not shown). After that, spermatozoa were further analyzed by using CASA technology, as described above, to confirm that the density gradient procedure did not change motility and morphology parameters. Then, sperm samples were treated in ice for 30 min with Somatic Cell Lysis Buffer (SCLB) (0.1% SDS, 0.5% Triton X-100 in DEPC-H_2_O) to eliminate any somatic cell contamination. Following the SCLB treatment and the microscope examination carried out to verify the elimination of somatic cells, an aliquot of sample was used to re-evaluate the number of live and motile spermatozoa under a light microscope in order to exclude effects on sperm vitality, motility as well as on sperm concentration, which is induced by the technical procedure (data not shown). Then, A-sperm and B-sperm samples were resuspended in sperm washing medium (HTF-IrvineScientific^®^) and counted under a light microscope using a Burker Chamber. The counting of 20 A-spermatozoa samples showed a media cell concentration of 6.5 x 10^6^ ± 0.5 × 10^6^, while the counting of 20 B-spermatozoa samples showed a media cell concentration of 11.5 × 10^6^ ± 0.5 × 10^6^. In order to pellet equal concentrations in both fractions for molecular investigations, 5 × 10^6^ cells were used. A-spermatozoa and B-spermatozoa pellets were stored at −80 °C.

### 2.4. Total RNA Preparation

Trizol^®^ Reagent (Invitrogen Life Technologies, Paisley, UK) was used to extract total RNA from human spermatozoa following the manufacturer’s instructions. The sample was homogenized in Trizol Reagent (1 mL Trizol^®^ Reagent/5 × 10^6^ sperm cells) and incubated for 5 min at 20 °C to permit nucleoprotein complexes dissociation. Then, 0.2 mL chloroform/mL Trizol^®^ Reagent was added and the sample was centrifuged at 12,000× *g* for 15 min at 4 °C. After centrifugation, the aqueous phase was transferred to a fresh tube and isopropyl alcohol (0.5 mL/mL Trizol^®^ Reagent) and 1 μL glycogen (20 mg/mL) were added to promote the precipitation of total RNA. After centrifugation at 12,000× *g* for 10 min at 4 °C, the RNA pellet was washed with 75% ethanol, centrifuged at 7500× *g* for 10 min at 4 °C and dissolved in an appropriate volume of DEPC treated water. By using a NanoDrop 2000 spectrophotometer (Thermo, Waltham, MA, USA), we assessed the quantity (ng/mL) and purity (260/280 and 260/230 ratios) of total RNAs. Then, the RNA aliquots (10 μg) were treated with 2U DNase I (RNase free DNase I, Ambion, Thermo Fisher Scientific, Massachusetts, USA) according to the manufacturer’s recommendations to remove potential contamination of genomic DNA. The RNAs were then preserved at −80 °C until the next step.

### 2.5. RNA Expression Analysis by One-Step Evagreen qRT-PCR

In accordance with the manufacturer’s instructions, we used the One-Step Evagreen qRT-PCR reaction kit containing qRT-PCR enzyme mix and an Evagreen qPCR Mastermix (Applied Biological Materials Inc., Richmond, BC, Canada) to investigate RNA expression. All reactions were performed by using 50 ng of total RNA on a CFX-96 Real Time PCR System (Biorad). Assays were carried out in triplicate and RNA expression was evaluated by using the CFX Manager software (Biorad). A negative control without RNA and a melting curve analysis in which all samples displayed single peaks for each primer pair were also included. *GAPDH* was used as reference gene for the normalization. QRT-PCR data for mRNA analysis were expressed by calculating delta-Cq values with the aim of expressing the pure expression profile, while qRT-PCR data for circRNA analysis were expressed as normalized fold expression by applying the 2^−∆∆Ct^ method.

### 2.6. PCR Primer Design

Primers for amplifying selected mRNAs and circRNAs in human spermatozoa samples were designed by using the online tool Primer-BLAST (http://www.ncbi.nlm.nih.gov/tools/primer-blast/, accessed on 28 July 2021). We also designed specific primers for the housekeeping gene used for normalization: *GAPDH* (glyceraldehyde 3-phosphate dehydrogenase). Primers for human genes are shown in Table 1.

### 2.7. Protein Extraction and Western Blot Analysis

Asthenozoospermic A-spermatozoa and B-spermatozoa were homogenized separately in RIPA buffer (PBS, pH 7.4, 10 mM dithiothreitol, 0.02% sodium azide, 0.1% SDS, 1% Nonidet P-40, 0.5% sodium deoxycholate and in the presence of protease inhibitors (10 μg/mL of leupeptin, aprotinin, pepstatin A, chymostatin and 5 μg/mL of TPCK)) and sonicated three times for 30 sec bursts, each at 60 mW. Proteins were separated by SDS-PAGE (8% acrylamide) and transferred to polyvinylidene difluoride membrane (GE Healthcare) at 280 mA for 2.5 h at 4 °C. The filters were treated for 2.5 h with a blocking solution (5% nonfat milk, 0.25% Tween-20 in Tris-buffered saline (TBS, pH 7.6)) and incubated with different primary antibody (CRISP2, diluted 1:500, sc 390914 Santa Cruz Biotechnology, Cambridge, UK; PATE1, diluted 1:500, ABC244 Sigma-Aldrich; CATSPER1, diluted 1:500, PA5-75788, Thermo Fisher; Tubulin, diluted 1:5000, Sigma-Aldrich (T5168); ERK2, diluted 1:500, sc-154 Santa Cruz Biotechnology, Cambridge, UK) in TBS-milk buffer (TBS pH 7.6, 3% nonfat milk) overnight, at 4 °C. The filters were washed in 0.25% Tween20-TBS and incubated with 1:1000 horseradish peroxidase-conjugated mouse IgG (Dako Corp., Milan, Italy) in TBS-milk buffer and then washed again. The enhanced chemiluminescence Western blotting detection system (Amersham ECL Western Blotting Detection Reagent, cod: RPN2106, GE Healthcare) was used to detect the immune complexes. Signals were quantified by densitometry analysis, adjusted relatively to Tubulin levels and graphed as OD fold change (mean ± S.E.M).

### 2.8. Asthenozoospermic Patient Treatment

In order to better understand if motility perturbations could be rooted in quantitative changes of transcripts in sperm, we carried out a pharmacological treatment of asthenozoospermic patients to improve sperm motility and this consisted of the oral administration of a mixture of amino acids, ornithine-citrulline-l-arginine, at a dose equal to 1000 mg of one capsule per day for 3 months. Such a treatment had a consolidated action on sperm vitality and motility, considering that amino acid supplementation strongly supports germ cell progression, improves antioxidant capacity and increases the secretion of both estradiol-17β and testosterone [41,43,44,45,46,47,48,49,50,51,52,53]. At the end of the therapy, semen parameters were evaluated to measure the improvement of sperm motility by using CASA technology, as described above. As reported in Table 2, a significantly increase (* *p* < 0.05) in the percentage of total sperm motility (approximately 20%) occurred following oral amino acid supplementation. In addition, pre-treated (pre-) and post-treated (post-) A-spermatozoa and B-spermatozoa collected from 20 patients (20 samples for each experimental group) were purified by the PureCeption gradient as described above. Subsequently, sperm samples were resuspended in PBS and counted under a light microscope using a Burker Chamber in order to pellet equal concentrations of spermatozoa (5 × 10^6^) used for subsequent molecular investigations.

### 2.9. Functional Annotation for circRNA/miRNA and Target miRNA Interaction

Based on both the TargetScan online analytical software (http://www.targetscan.org, accessed on 28 July 2021) and Arraystar’s miRNA target prediction software, the circRNA/miRNA interaction was predicted for all DE-circRNAs. Validated and predicted targets of miRNAs were retrieved by Diana TarBase 8.0 (http://www.microrna.gr/tarbase, accessed on 28 July 2021); the circRNA/miRNA/Target network (ceRNET) was built and visualized by using the Bisogenet plug-in of Cytoscape (www.cytoscape.org, accessed on 28 July 2021).

### 2.10. Statistical Analysis

Student’s *t*-test (for two independent group comparison) and ANOVA followed by Tukey’s post hoc *t*-test (for multigroup comparison) (Prism 5.0, GraphPad Software (San Diego, CA, USA)) were conducted to identify groups having different mean. Differences with *p* < 0.05 were considered statistically significant and the data were expressed as the mean ± S.E.M.

## 3. Results

### 3.1. Expression of CATSPER1, PATE1 and CRISP2 in Normozoospermic Sperm

**Figure 1:**
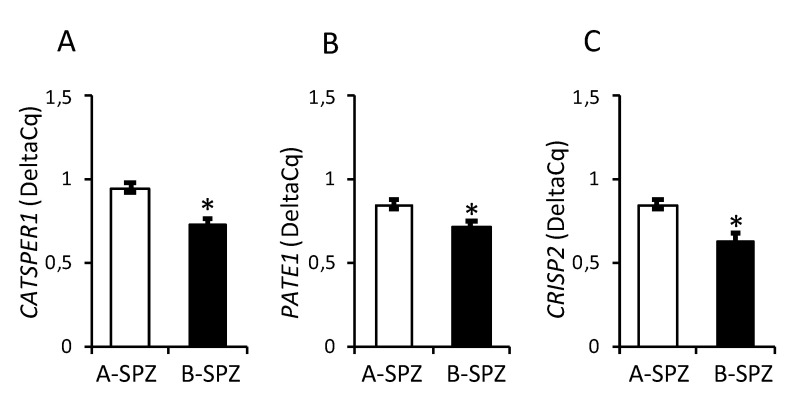
Expression of *CATSPER1*, *PATE1* and *CRISP2* in Normozoospermic Sperm. (**A**–**C**) Differential expression analysis of mRNAs between A-spermatozoa and B-spermatozoa (A-SPZ; B-SPZ) collected from normozoospermic volunteers by qRT-PCR. (**A**) *CATSPER1*, (**B**) *PATE1* and (**C**) *CRISP2* were normalized by using *GAPDH* as the reference gene and data are expressed as normalized quantification cycle for PCR (Delta-Cq). All data are reported as mean value ± S.E.M; *: *p* < 0.05.

In order to evaluate a putative correlation between the expression levels of *CATSPER1*, *PATE1* and *CRISP2* mRNAs and sperm motility in physiological conditions, sperm samples from normozoospermic volunteers were processed to separate A-spermatozoa and B-spermatozoa fractions (A-SPZ; B-SPZ) on the basis of morphology and motility parameters. Thus, the two sperm fractions were used to analyze *CATSPER1*, *PATE1* and *CRISP2* mRNA levels by qRT-PCR. Results showed a significant reduction in all three mRNAs in B-spermatozoa compared to A-spermatozoa (*p* < 0.05, Figure 1A–C), thus, suggesting a possible correlation between their levels of expression and the low motility of B-spermatozoa.

### 3.2. Expression of CATSPER1, PATE1, CRISP2 and circRNAs in Asthenozoospermic Sperm

**Figure 2:**
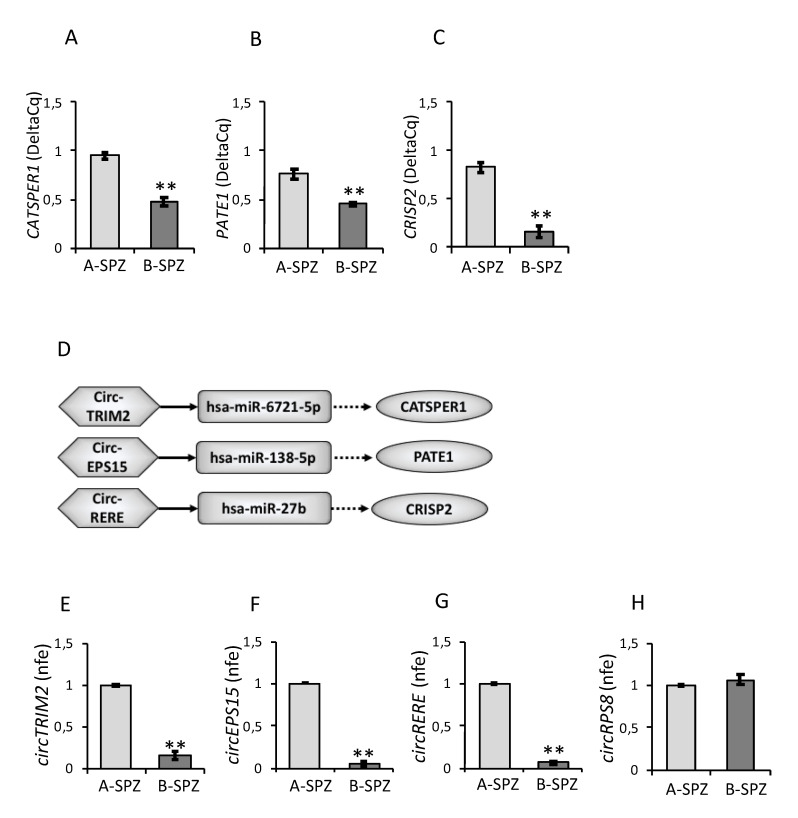
Expression of *CATSPER1*, *PATE1*, *CRISP2* and circRNAs in Asthenozoospermic Sperm**.** (**A**–**C**) Differential expression analysis of mRNAs between A-spermatozoa and B-spermatozoa (A-SPZ; B-SPZ) collected from asthenozoospermic patients by qRT-PCR. (**A**) *CATSPER1*, (**B**) *PATE1* and (**C**) *CRISP2* were normalized by using *GAPDH* as reference. Data are expressed as normalized quantification cycle for PCR (Delta-Cq) and reported as mean value ± S.E.M; **: *p* < 0.01. (**D**) Functional clustering of circRNAs upregulated in asthenozoospermic A-spermatozoa identified by the microarray approach [41]. Three circRNAs tether a group of miRNAs involved in *CATSPER1*, *PATE1* and *CRISP2* mRNA regulation. Networks were built using Cytoscape. Hexagonal and rectangular symbols represent circRNAs and miRNAs, respectively, while the elliptic symbol represents mRNA target. The arrow indicates the tethering activity of circRNAs towards miRNAs, while the dotted arrow indicates the inhibiting activity of miRNAs towards mRNAs. (**E**–**H**) Differential expression analysis of circRNAs between A-spermatozoa and B-spermatozoa collected from asthenozoospermic patients by qRT-PCR. (**E**) circTRIM2, (**F**) circEPS15, (**G**) circRERE (**H**) and circRPS8 were normalized by using *GAPDH* as reference and expressed as normalized fold expression (nfe). All data are reported as mean value ± S.E.M; **: *p* < 0.01.

Sperm samples from asthenozoospermic patients were processed to analyze *CATSPER1*, *PATE1* and *CRISP2* mRNA levels separately in A-spermatozoa and B-spermatozoa (A-SPZ; B-SPZ) by qRT-PCR. A significant reduction in all three mRNAs was detected in B-spermatozoa compared to A-spermatozoa (*p* < 0.01, Figure 2A–C), strongly confirming a correlation between their levels of expression and the low motility of B-spermatozoa, as previously shown in normozoospermic A-spermatozoa and B-spermatozoa.

Since RNAs are able to reciprocally interact forming complicate networks, we evaluated if *CATSPER1*, *PATE1* and *CRISP2* mRNA downregulation may depend on an inhibitory upstream RNA network. To this end, by using a bioinformatic approach (www.targetscan.org), we identified three miRNAs—hsa-miR-6721-5p, hsa-miR-138-5p and hsa-miR-27b—able to target *CATSPER1*, *PATE1* and *CRISP2* mRNAs, respectively. Subsequently, the identified miRNAs were matched with our DE-circRNAs array dataset and carried out on asthenozoospermic A-spermatozoa and B-spermatozoa separately [41] in order to identify the putative circRNAs that are downregulated in B-spermatozoa and involved in the regulation of *CATSPER1*, *PATE1* and *CRISP2* expression levels. The construction of the relative ceRNET is reported in Figure 2D.

A differential expression profile of circTRIM2, circEPS15 and circRERE was demonstrated in A-spermatozoa and B-spermatozoa of asthenozoospermic patients by qRT-PCR analysis. All circRNAs analyzed were significantly lower in B-spermatozoa compared to A-spermatozoa (*p* < 0.01) (Figure 2E–G). As an internal control for circRNA content, we evaluated circRPS8 that was chosen from our circRNA dataset in asthenozoospermic spermatozoa as not significantly changing between A-spermatozoa and B-spermatozoa. CircRPS8 appeared constant in expression (Figure 2H) and did not target any of the miRNAs previously identified.

### 3.3. Expression of CATSPER1, PATE1, CRISP2 and circRNAs in Asthenozoospermic Sperm after an Oral Amino Acid Supplementation

**Figure 3:**
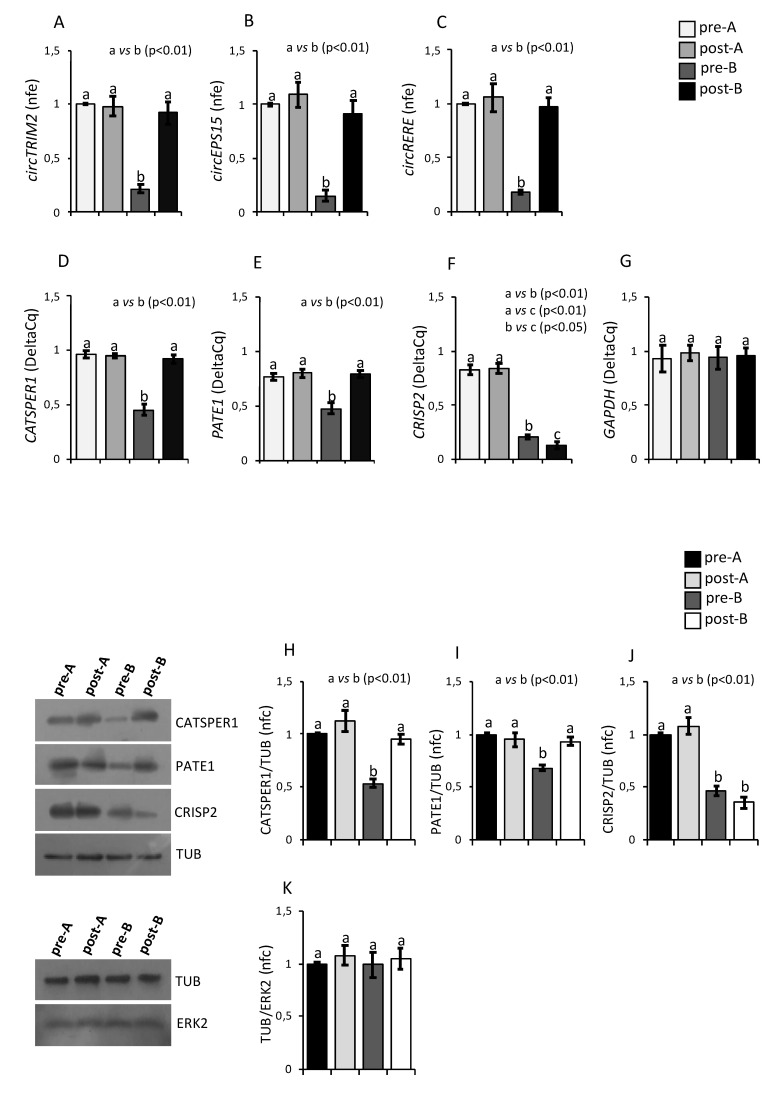
Expression of CATSPER1, PATE1, CRISP2 and circRNAs in Asthenozoospermic Sperm after an oral Amino Acid Supplementation. (**A**–**F**) Expression of circRNAs and mRNAs in A-spermatozoa and B-spermatozoa collected from asthenozoospermic patients before (pre-A; pre-B) and after (post-A; post-B) an oral amino acid supplementation therapy by qRT-PCR. (**A**) circTRIM2, (**B**) circEPS15, (**C**) circRERE, (**D**) *CATSPER1*, (**E**) *PATE1* and (**F**) *CRISP2* were normalized by using *GAPDH* as the reference gene. (**G**) Expression of *GAPDH* mRNAs in A-spermatozoa and B-spermatozoa collected from asthenozoospermic patients before (pre-A; pre-B) and after (post-A; post-B) an oral amino acid supplementation therapy by qRT-PCR. *GAPDH* was normalized by using *PCNA* as the reference gene. CircRNAs data were expressed as normalized fold expression (n.f.e.); mRNAs data were expressed as normalized quantification cycle for PCR (Delta-Cq). All data were reported as mean value ± S.E.M. Experimental groups with statistically significant differences (*p* < 0.05; *p* < 0.01) were indicated with different letters; the experimental groups without statistically significant differences were indicated with the same letter. (**H**–**K**) Western blot analysis of CATSPER1 (**H**), PATE1 (**I**), CRISP2 (**J**) and Tubulin (**K**) proteins in A-spermatozoa and B-spermatozoa collected from asthenozoospermic patients before (pre-A; pre-B) and after (post-A; post-B) an oral amino acid supplementation therapy. In (**H**–**J**), signals were quantified by densitometry analysis and normalized against Tubulin. In (**K**), Tubulin signals were quantified by densitometry analysis and normalized against ERK2. Data were expressed as normalized fold change (n.f.c) and reported as mean ± SEM. Experimental groups with statistically significant differences (*p* < 0.01) were indicated with different letters; the experimental groups without statistically significant differences were indicated with the same letter.

With the aim to evaluate if an oral amino acid supplementation used to improve sperm motility in asthenozoospermic patients may affect circRNA and mRNA content in A-spermatozoa and B-spermatozoa, we analyzed the expression levels of circTRIM2, circEPS15 and circRERE in pre-treated (pre-) and post-treated (post-) A-spermatozoa and B-spermatozoa, simultaneously. Results showed that the pharmacological treatment did not affect circRNA levels in post-A spermatozoa compared to pre-A spermatozoa (Figure 3A–C). Interestingly, all circRNAs downregulated in pre-B spermatozoa were restored to pre-A levels following the oral amino acid supplementation (post-B spermatozoa) (*p* < 0.01, Figure 3A–C).

Analogously, we analyzed the expression levels of *CATSPER1*, *PATE1* and *CRISP2* in pre-treated (pre-) and post-treated (post-) A-spermatozoa and B-spermatozoa by qRT-PCR analysis. Results showed that *CATSPER1* and *PATE1* significantly increased in post-B compared to pre-B spermatozoa (*p* < 0.01, Figure 3D,E), while *CRISP2* significantly decreased following the pharmacological treatment (*p* < 0.05, Figure 3F). Instead, as observed for circRNA levels, there were no significant differences for *CATSPER1*, *PATE1* and *CRISP2* levels between pre-A and post-A spermatozoa (Figure 3D–F).

Since GAPDH enzyme is involved in glycolytic pathway that seems to participate to sperm motility regulation, it is plausible to ask if *GAPDH* may be a good reference gene in our experimental groups. For this reason, its expression was assessed against *PCNA* and no significant changes were observed (Figure 3G). This profile well fitted with that suggested by Paoli et al. (2017) [54] that investigated *GAPDH* expression in human sperm in order to evaluate its potential correlation with asthenozoospermia. Since no changes were observed in that case as in our experimental groups, it is conceivable to sustain their hypothesis that sperm hypomotility may be due to a possible post-transcriptional impairment of the control mechanism, such as mRNA splicing, or to post-translational changes rather than to a direct transcriptional dysregulation of *GAPDH* [54].

In order to investigate if the expression levels of *CATSPER1, PATE1* and *CRISP2* mRNAs well matched the protein profiles, Western blot analysis was carried out in pre-treated (pre-) and post-treated (post-) A-spermatozoa and B-spermatozoa of asthenozoospermic patients, simultaneously. As shown, no significant effect was observed in A-spermatozoa fraction (post-A spermatozoa) following the pharmacological treatment compared to pre-A spermatozoa (Figure 3H–J). Interestingly, CATSPER1 and PATE1 protein levels, which were significantly lower in pre-B than pre-A spermatozoa (*p* < 0.01), were restored to pre-A spermatozoa levels following the oral amino acid supplementation (*p* < 0.01, Figure 3H–I). Conversely, CRISP2 protein levels were not fully restored in post-B spermatozoa (Figure 3J). In order to make sure that Tubulin protein levels were constant in all experimental groups, Western blot analysis was carried out in pre-treated (pre-) and post-treated (post-) A-spermatozoa and B-spermatozoa of asthenozoospermic patients by using the ERK2 protein as reference. As shown in Figure 3K, Tubulin levels were constant in all sperm populations, excluding any artifacts. These results are in accordance with data by Chawan et al. (2020) [55] in which sperm motility is rather influenced by post-translational changes in microtubule associated proteins [55].

### 3.4. Expression of circSEPT10 in Asthenozoospermic Patients

**Figure 4:**
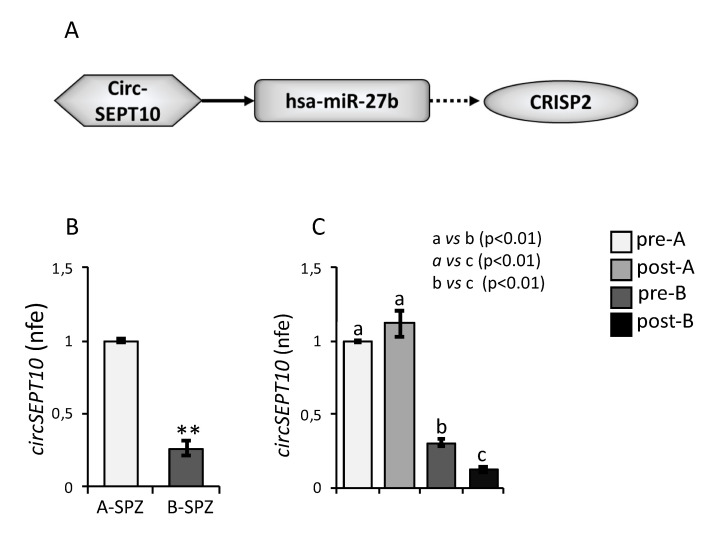
Expression of circSEPT10 in Asthenozoospermic Patients. (**A**) Functional clustering of one circRNA upregulated in asthenozoospermic A-spermatozoa. CircSEPT10 tethers a miRNA involved in *CRISP2* mRNA regulation. Networks were built using Cytoscape. Hexagonal and rectangular symbols represent circRNAs and miRNAs, respectively, while the elliptic symbol represents the mRNA target. The arrow indicates the tethering activity of circSEPT10 toward miRNA, while the dotted arrow indicates the inhibiting activity of miRNAs toward *CRISP2*. (**B**) Differential expression analysis of circSEPT10 between A-spermatozoa and B-spermatozoa collected from asthenozoospermic patients by qRT-PCR. (**C**) Expression of circSEPT10 in A-spermatozoa and B-spermatozoa collected from asthenozoospermic patients before (pre-A; pre-B) and after (post-A; post-B) an oral amino acid supplementation therapy. All qRT-PCR data were normalized by using *GAPDH*, expressed as normalized fold expression (n.f.e.) and reported as mean value ± S.E.M. Experimental groups with statistically significant differences (*p* < 0.01) were indicated with different letters; the experimental groups without statistically significant differences were indicated with the same letter. **: *p* < 0.01.

Since the pharmacological treatment significantly restored *CATSPER1* and *PATE1* mRNA levels while *CRISP2* decreased even if circRERE—the potential circRNA upstream of *CRISP2* mRNA—significantly increased, we tried to deeply examine the ceRNET regulating *CRISP2* mRNA by bioinformatic analysis. Thus, we pointed to another circRNA (circSEPT10) involved in hsa-miR-27b regulation (Figure 4A). The analysis of circSEPT10 levels in asthenozoospermic spermatozoa by qRT-PCR showed a significant reduction in B-spermatozoa compared to A-spermatozoa (*p* < 0.01, Figure 4B).

Interestingly, the analysis of circSEPT10 levels in pre-treated (pre-) and post-treated (post-) A-spermatozoa and B-spermatozoa of asthenozoospermic patients showed that circSEPT10 levels in post-B spermatozoa were lower than pre-B spermatozoa (*p* < 0.01), suggesting a partial restoration of the circRNA content (Figure 4C), while no effect was observed in post-A spermatozoa following the pharmacological treatment (Figure 4C).

## 4. Discussion

Sperm cells are more than just a vehicle of a haploid genome. During epididymal transit, they enrich themselves with a large repertoire of molecules—both transcripts and proteins—that are received from the epithelial epididymal cells through epididymosomes [1,55,56]. This cargo has been related to sperm quality and infertility onset, as well as hypothesized to be involved in downstream post-fertilization development events [57,58,59,60]. Sperm molecular signature appears dynamically shaped by paternal experiences and inherited from the offspring through a mechanism known as intergenerational (if information is passed between two generations) or transgenerational (if information is passed across multiple generations, usually three or more) epigenetic inheritance [61,62,63].

One of the most intriguing query is related to a possible correlation between sperm RNA profile and male infertility; this aspect concerns both coding and non-coding RNAs (ncRNAs) [63]. The biological significance of sperm derived mRNAs is still debated due to the dormant transcriptional state of these cells as well as the lack of the molecular cytoplasmic machinery supporting their translatability. A possible de novo translation of sperm derived mRNAs has been suggested or, alternatively, sperm derived mRNAs may have a role in sperm in their own guise. Here, we suggest that sperm mRNA cargo may shed light on sperm quality.

Despite the advances in the etiology of male infertility, detailed molecular mechanisms underlying asthenozoospermia have yet to be deeply unraveled.

By using a microarray strategy, differentially abundant transcripts have been identified in infertile patients when compared with fertile controls. The deregulated transcripts are involved in spermatogenesis, sperm motility, DNA repair, oxidative stress regulation and histone modifications [18,64,65].

Nowadays, bioinformatic and transcriptomic approaches are moving towards the identification of complicated regulatory networks governing the biogenesis, the stability, the functional role of RNAs and the translatability of mRNAs. Special actors in these networks are ncRNAs, mainly miRNAs, long ncRNAs and circRNAs [66]. All these molecules actively take part to the wide repertoire of DE-RNAs in infertile men.

The role of miRNAs in physiological processes such as cell differentiation and developmental timings has been deeply investigated [67]; their deregulation has been linked to male infertility [68] and widely detailed in seminal plasma and spermatozoa of asthenozoospermic patients [69,70,71].

In the scenario of ncRNAs, an increasing interest has been addressed towards circRNAs. Their expression has been analyzed in testis, seminal plasma and spermatozoa [35,36,37] in both normozoospermic and infertile patients [39,41] and correlated with sperm motility and quality [41,72]. CircRNAs are actively involved in the regulation of mitochondrial functions and are differentially expressed in sperm collected from asthenozoospermic patients; they are modulated by pharmacological treatments able to improve sperm motility, strengthening the suggested diagnostic power of these molecules [41].

Recently, the critical role of circRNAs in testis physiology and sperm motility has been reinforced by using a knockout strategy and, lastly, by counting circBOULE among the DE-circRNAs in asthenozoospermic patients [73].

Starting from this evidence, we sought to shed light on the potential molecular mechanisms responsible for the transcriptional deregulation in asthenozoospermic sperm, focusing on *CATSPER1*, *PATE1* and *CRISP2* mRNAs, which are known to be involved in the regulation of sperm motility [19,20,21,27,74] and for which their differential expression was related here to sperm quality. In fact, their low expression, both at the transcript and protein levels in B-spermatozoa, a sperm population of low quality, strongly supported such a hypothesis.

Since the demonstration that RNAs are able to physically and functionally communicate each other through networks, we decided to better understand whether upstream of the differential expression levels of *CATSPER1*, *PATE1* and *CRISP2* mRNAs were involved circRNAs. With this in mind, we identified three miRNAs—hsa-miR-6721-5p, hsa-miR-138-5p and hsa-miR-27b—able to downstream target *CATSPER1*, *PATE1* and *CRISP2* mRNAs, respectively, and matched them with our DE-circRNAs array dataset separately carried out on asthenozoospermic A-spermatozoa and B-spermatozoa [41]. The putative involved circRNAs were then analyzed. Accordingly to the levels of mRNA targets, circTRIM2, circEPS15 and circRERE—upstream of *CATSPER1*, *PATE1* and *CRISP2* mRNAs, respectively—were found significantly downregulated in B-spermatozoa of asthenozoospermic patients.

As previously suggested, the observation of quantitative changes in the expression of sperm derived mRNAs and/or ncRNAs [18,75] may provide molecular information concerning sperm quality [62,63,76,77]. Accordingly, a possible modulation of their expression after pharmacological treatments, consisting in the oral administration of a mixture of amino acids, ornithine-citrulline-L-arginine, used to improve sperm motility in asthenozoospermic B-spermatozoa, may be an interesting aspect supporting the hypothesis of the involvement of these molecules in the control of sperm physiology. Among the constituents of the mixture, L-arginine has a consolidated role in stimulating sperm motility in several species [78], increasing the rate of glycolysis with higher rates of ATP and lactate generation in spermatozoa and with beneficial effects linked to nitric oxide (NO) production [79].

Therefore, the effect of the oral amino acid supplementation has been observed on A-spermatozoa and B-spermatozoa. The expression of *CATSPER1* and *PATE1* was significantly restored in B-spermatozoa after the treatment, whereas *CRISP2* expression decreased. Analogously, we analyzed circTRIM2, circEPS15 and circRERE levels in B-spermatozoa, pre-treatment and post-treatment and all circRNAs—downregulated in pre-B spermatozoa—significantly increased after the pharmacological treatment; this includes circRERE—the potential circRNA upstream of *CRISP2* mRNA. This result did not support the decrease in *CRISP2* expression.

The expression levels of circRNAs and mRNAs did not change between pre-A and post-A spermatozoa, suggesting that most of the effects were related to B-spermatozoa fraction.

In addition, CATSPER1 and PATE1 protein profiles were reverted in post-B spermatozoa, well matching mRNA profiles. Conversely, a not restored level was observed for CRISP2 protein. Similar to mRNAs, protein profiles did not significantly change between pre-A and post-A spermatozoa.

In normozoospermic volunteers, circRNAs have been shown to organize themselves in functional clustering in order to target the same group of miRNAs [40]. With this in mind, we deeply examined the ceRNET regulating *CRISP2* mRNA by a bioinformatic analysis in order to point to another circRNA (circSEPT10) involved in hsa-miR-27b regulation. The candidate was circSEPT10 that, similarly to circRERE, was upregulated in A-spermatozoa vs. B-spermatozoa. However, after the pharmacological treatment, circSEPT10 levels in B-spermatozoa were lower than in pre-treated control spermatozoa, thus, suggesting a not full restoration of the transcriptional levels. This result was also well in line with a partial recovery of sperm motility observed in the patient cohort.

In conclusion, data shown here suggest a potential differential cargo of molecules, both mRNAs and proteins, in high quality and low quality spermatozoa collected from asthenozoospermic patients.

A ceRNET dependent modulation was also investigated, suggesting an intriguing role of circRNAs in driving molecular mechanisms on the basis of sperm motility.

Much effort should be conducted in shedding light on the fate of sperm derived circRNAs and mRNAs once transferred to the oocyte upon fertilization. This aspect still remains under investigation and potentially may help to define several features of the offspring, including its health and fertility.

## Figures and Tables

**Table 1:** Primer Sequence and Annealing Temperature for mRNAs and circRNAs.

Gene Primers	Sequences 5′-3′	Tm (°C)
*CRISP2* S	TGCCATTATTGTCCTGCTGGT	56
*CRISP2* AS	CATGTTCACAGCCAGTTGTATTCT	
*CATSPER1* S	AAGGGCAATTTCAGAAACGCA	57
*CATSPER1* AS	TCAAAGGCCAAGGATTGGGTTA	
*PATE1* S	TCTGCTGCTTTAGGGCGTTAT	57
*PATE1* AS	GGTGGCACATCCTACACYGA	
*GAPDH* S	TGCACCACCAACTGCTTAGC	58
*GAPDH* AS	GGCATGGACTGTGGTCATGAG	
*PCNA* S	TAAACCTGCAGAGCATGGAC	53
*PCNA* AS	GCCGGCGCATTTTAGTATTT	
*circRERE* S	CAGACCCAGTTATCAAGAACCGA	54
*circRERE* AS	GGGAGTTGTGGACCTAAGGG	
*circEPS15* S	CCTTTTGTTGGCAATCTCTTCTC	52
*circEPS15* AS	CGGCTCAGCTCTTCTCTAGC	
*circTRIM2* S	TTGCCCAAACCACGATG	52
*circTRIM2* AS	ACAGGACTTGGGATGTTGG	
*circSEPT10* S	ACCCATACCAGGCACTATGA	52
*circSEPT10* AS	TGAAAGAGCTGACTGGCTTG	
*circRPS8* S	GTTGTGGCCGTCTTGGTCAC	58
*circRPS8* AS	GGAGAGCAAGGCAAGTGAGG	

**Table 2:** Parameters evaluated in asthenozoospermic patients (*n* = 20), pre-treatment and post-treatment; *: *p* < 0.05.

Parameters Studied	Asthenozoospermic Men Pre-Treatment	Asthenozoospermic Men Post-Treatment
Age of patients	28.63 ± 4.3	29.61 ± 5.8
Semen total volume (mL)	2.35 ± 0.44	2.74 ± 0.62
Sperm concentration (×10^6^/mL)	31.32 ± 10.31	33.75 ± 11.82
Total motility (%)	23.21 ± 1.38	43.45 ± 2.42 *
Progressive motility (%)	20.35 ± 2.27	28.73 ± 1.27 *
Sperm vitality (%)	41.34 ± 5.32	46.92 ± 3.25
Normal morphology (%)	12.5 ± 3.3	15.9 ± 4.5
